# Drought stress in *Cannabis sativa* L.: current knowledge, research gaps, and future perspectives – a review

**DOI:** 10.3389/fpls.2026.1890092

**Published:** 2026-07-27

**Authors:** Saber Delpasand Khabbazi, Sang-Hyuck Park, Byeong-Ryeol Ryu, Jose F. Da Cunha Leme Filho, Güngör Yılmaz, Afsaneh Delpasand Khabbazi, Deepali Singh, Sachin Teotia

**Affiliations:** 1 Department of Agriculture and Food, Institute of Hemp Research, Yozgat Bozok University, Yozgat, Türkiye; 2 Institute of Cannabis Research, Colorado State University Pueblo, Pueblo, CO, United States; 3 School of Biological Sciences, Southern Illinois University, Carbondale, IL, United States; 4 School of Forestry and Horticulture, Southern Illinois University, Carbondale, IL, United States; 5 Department of Field Crops, Faculty of Agriculture, Yozgat Bozok University, Yozgat, Türkiye; 6 School of Biotechnology, Gautam Buddha University, Greater Noida, India; 7 Department of Life Sciences, J.C. Bose University of Science and Technology (YMCA), Faridabad, Haryana, India; 8 Department of Biotechnology, Sharda University, Greater Noida, India

**Keywords:** *Cannabis sativa*, drought stress, molecular regulation, morphophysiological responses, plant–microbe interactions, secondary metabolites, sustainable agriculture

## Abstract

Cannabis (*Cannabis sativa* L.) has gained increasing agronomic, industrial, and therapeutic importance owing to its fiber, seed, and bioactive cannabinoid content. However, water limitation represents a major environmental constraint affecting cannabis growth, yield, and phytochemical composition. The increasing frequency and severity of drought events associated with climate change pose a growing challenge to agricultural sustainability, highlighting the need for improved crop performance under water-limited conditions. In this context, understanding plant responses to water deficit is essential for enhancing drought tolerance and maintaining productivity. Although recent studies have expanded our understanding of cannabis responses to drought, important knowledge gaps still exist regarding the mechanisms underlying drought adaptation. Current evidence indicates that cannabis responds to water limitation through coordinated changes in growth, physiology, secondary metabolism, molecular regulation, and rhizosphere interactions. Nevertheless, several key aspects, particularly root-mediated adaptation, RNA-mediated regulation, and epigenetic control, are still insufficiently understood. This review integrates current knowledge of cannabis drought responses from morphophysiological, molecular, and plant–microbe interaction perspectives, highlights major research gaps, and proposes future directions for improving drought resilience in cannabis cultivation.

## 1 Introduction

*Cannabis sativa* L. (hereafter referred to as cannabis throughout this review) is a versatile member of the Cannabaceae family, native to Central Asia, and was cultivated for centuries before experiencing a prolonged period of legal restrictions (McPartland et al., 2019). It is among the earliest domesticated multipurpose crops and was historically valued mainly for its fiber and seeds (Ren et al., 2021). During the twentieth century, cannabis cultivation declined markedly due to the expansion of cotton production and the widespread adoption of synthetic fibers (Salentijn et al., 2015). In addition, psychoactive properties and associated legal restrictions further limited its production in many countries (Schlosser, 1994). However, cannabis cultivation never disappeared entirely, persisting at low levels in some regions. Following the relaxation of regulatory restrictions, cannabis cultivation and breeding have reemerged as areas of significant scientific and industrial interest, particularly for sustainable raw material production, bio-based products, and medicinal applications (Cherney and Small, 2016). Today, cannabis is increasingly cultivated for industrial, nutritional, and medicinal purposes, and its therapeutic potential is largely attributed to its diverse cannabinoid profile. Based on cannabinoid composition, cannabis is commonly classified into Δ9-tetrahydrocannabinol (THC)-type, hybrid, and cannabidiol (CBD)-type chemotypes while (Galal et al., 2009; National Academies of Sciences, 2017). In general, THC-type cannabis is regarded as drug-type cannabis, whereas CBD-dominant and low-THC types are classified as industrial hemp cultivated for fiber and seed production.

Given its increasing economic and medicinal importance, understanding how cannabis responds to major environmental stresses has become increasingly important. Drought stress represents one of the most serious environmental constraints affecting agricultural productivity. Its increasing frequency and intensity under climate change pose significant risks, particularly in arid and semi-arid regions (OECD, 2025). Yield reductions during drought years can exceed 20%, which can have significant consequences for food security and agricultural sustainability. Among the various abiotic stresses affecting cannabis production, drought is particularly important because it directly influences plant growth, biomass accumulation, and secondary metabolism, thereby affecting both crop productivity and quality (Sharma et al., 2025; Gill et al., 2025, 2022). Considering the growing economic and industrial relevance of cannabis, understanding how water deficit affects its growth, yield, and secondary metabolism has become increasingly important (Zimniewska, 2022). Furthermore, increasing water scarcity makes it essential to better understand drought responses in cannabis and develop more resilient cultivation strategies. Compared with other fiber crops such as cotton (*Gossypium hirsutum* L.), fiber-type cannabis or industrial hemp requires substantially lower water inputs, with reported reductions of approximately 60% in water footprint and 84% in crop irrigation requirement (Wise et al., 2023). Nevertheless, its productivity can decline under water deficit, particularly during critical phenological stages, with responses further shaped by stress severity and genotype (Gill et al., 2022; Morgan et al., 2024).

Plant responses to drought involve coordinated adjustments at morphological, physiological, biochemical, and molecular levels. Common adaptive responses include stomatal regulation, modification of leaf area, enhanced root development, and osmotic adjustment through compatible solutes (Farooq et al., 2012; Osakabe et al., 2014). These responses can also be modulated by plant-associated beneficial microorganisms, which may enhance drought tolerance through improved water relations and stress regulation (Dong et al., 2019; Ryu et al., 2025). At the molecular level, drought stress can alter secondary metabolism, including phenolic accumulation and, in cannabis, cannabinoid composition (Sharma et al., 2025). Epigenetic mechanisms, such as DNA methylation and histone modifications, have been associated with stress memory and enhanced tolerance upon repeated exposure (Liu and He, 2020). Non-coding RNAs (ncRNAs), particularly microRNAs (miRNAs), also contribute to drought responses by regulating stress-related gene expression and developmental processes (Bolc et al., 2025). Although molecular studies in cannabis are limited, evidence from other crop species suggests that RNA-mediated and epigenetic regulatory mechanisms may also play a role in cannabis drought adaptation.

Given the complexity of plant responses to drought across multiple biological levels, understanding the underlying regulatory mechanisms is essential for improving stress tolerance. Such knowledge, together with advanced genetic engineering approaches, can facilitate the development of crop varieties with enhanced stress tolerance and improved agronomic performance (Khabbazi et al., 2020, 2021). This review provides an integrated overview of how cannabis responds to water deficit at multiple levels, ranging from morphological and physiological adjustments to biochemical and molecular responses, and discusses the effects of drought on secondary metabolites and molecular regulators, as well as the role of beneficial microorganisms in enhancing drought tolerance in cannabis. We identify key knowledge gaps and propose future research directions aimed at improving drought resilience in this economically and industrially important crop.

## 2 Methodology

This review was conducted as a narrative review focusing on drought stress responses in cannabis. The literature search was performed using the Web of Science, Scopus, and Google Scholar databases. Searches were carried out at different time points between September 2025 and April 2026 to include recently published studies. The search strategy used combinations of keywords including “*Cannabis sativa*”, “hemp”, “drought stress”, “water deficit”, “abiotic stress”, “root system architecture”, “water use efficiency”, “photosynthesis”, “abscisic acid”, “secondary metabolism”, “cannabinoids”, “glandular trichomes”, “epigenetics”, “DNA methylation”, “non-coding RNAs”, and “microbiome”. Only peer-reviewed articles published in English were included. Relevant review articles, book chapters indexed in Web of Science or Scopus, and authoritative reports identified through Google Scholar were also considered when they were directly related to the scope of this review. Following the literature search, 191 references were selected based on their relevance to the scope of this review. Studies focusing on drought responses in cannabis were prioritized, whereas evidence from other plant species was included when it provided mechanistic insights relevant to drought stress in cannabis.

## 3 Morphophysiological responses of cannabis to drought stress

Water limitation induces morphological and physiological adjustments that affect plant productivity and survival under water deficit conditions. These responses include changes in stomatal behavior, osmotic adjustment, photosynthesis, water-use efficiency (WUE), growth, and biomass allocation. Together, they reflect a strategy of conservative water use, rapid physiological responses, and the maintenance of reproductive capacity despite reduced vegetative growth (Gill et al., 2025, 2022). In cannabis, these responses are shaped by interacting structural and functional traits, resulting in complex and genotype-specific adaptations with important implications for breeding and water management. This variability was demonstrated under controlled rain-out shelter conditions, where three genetically diverse cannabis genotypes exposed to well-watered (75% field capacity), moderate drought (40% field capacity), and severe drought (0% field capacity) exhibited contrasting physiological responses. Some genotypes maintain higher photosynthetic carbon assimilation and photosystem II efficiency under stress, whereas others rely on enhanced non-photochemical quenching and proline accumulation, or adopt more conservative water-use strategies (Mateva et al., 2024).

### 3.1 Growth and morphological adjustments

Water limitation substantially alters cannabis morphology, primarily through reductions in vegetative growth parameters. Under moderate and severe water deficit conditions, significant decreases in shoot biomass, root biomass, plant height, and leaf area have been observed, indicating that growth inhibition is a central component of drought response (Farooq et al., 2009; Morgan et al., 2024). In controlled greenhouse experiments, using the cannabis cultivar “Black Label” grown in free-draining pots under a 26/18 °C day/night thermoperiod with 12/12 h day/night photoperiod, moderate water deficit reduced shoot dry weight by approximately 66%, while severe deficit resulted in a reduction of about 96% compared with well-watered plants. Root dry weight also declined markedly, with reductions of approximately 45% under moderate stress and up to 95% under severe water deficit (Gill et al., 2022). Similarly, greenhouse experiments conducted with the cannabis cultivars “BaOx” and “Cherry Mom” under controlled irrigation regimes (70–100% field capacity for well-watered plants, followed by moderate and severe drought treatments during flowering). Severe drought reduced biomass by approximately 44–47% in sensitive genotypes, whereas other genotypes showed little to no significant response under similar stress conditions, with overall biomass losses reaching up to 54% under high-intensity drought (Morgan et al., 2024). These pronounced reductions reflect the sensitivity of vegetative growth to soil water availability. At the molecular level, transcriptomic analysis of the cannabis cultivar “Hanma No. 2” subjected to progressive drought stress revealed coordinated regulation of genes involved in photosynthesis, chlorophyll metabolism, carbon and nitrogen metabolism, and plant hormone signaling, indicating that growth inhibition is accompanied by extensive metabolic reprogramming rather than being solely a consequence of reduced water availability (Jiang et al., 2021). Decreased plant height and leaf area under water deficit further demonstrate morphological adjustment toward limiting the transpiring surface. Reduced leaf expansion and canopy size likely contribute to decreased evaporative demand, thereby enhancing water conservation. This morphological plasticity is consistent with drought avoidance strategies observed in other crops, where growth limitation functions to balance water uptake capacity with transpiration load (Jaleel et al., 2009).

Once established, cannabis exhibits a moderate degree of tolerance to water-limited conditions; however, severe drought induces early maturation and stunted growth (Adesina et al., 2020). Under extreme water deficit, total seed yield can decline drastically (by ~95–98%), primarily due to a marked reduction in seed number per plant, while seed size-related traits, including seed area, 100-seed weight, and seed shape parameters, remain largely stable (Berrada, 2019; Gill et al., 2022). This indicates that drought predominantly constrains seed formation rather than individual seed development or filling, with plants maintaining the production of fully developed seeds even under severe stress. Additionally, cannabis can withstand prolonged water limitation, remaining viable for up to 85 days under severe drought conditions in greenhouse conditions (Gill et al., 2022). Collectively, these responses suggest a stress-adaptive shift in resource allocation, whereby reproductive quality is maintained at the expense of quantity, consistent with a bet-hedging strategy that may enhance reproductive assurance under fluctuating water availability.

### 3.2 Stomatal regulation and integrated drought responses

Stomata are microscopic pores in the leaf epidermis that regulate CO_2_ uptake and water vapor loss, thereby controlling the balance between carbon assimilation and transpiration. This regulation is central to WUE and plant stress resilience (Bertolino et al., 2019; Haworth et al., 2021). Stomatal conductance is governed by structural traits, including stomatal size and density, and by physiological control of guard cell aperture (Lawson and Matthews, 2020).

**Figure 1:**
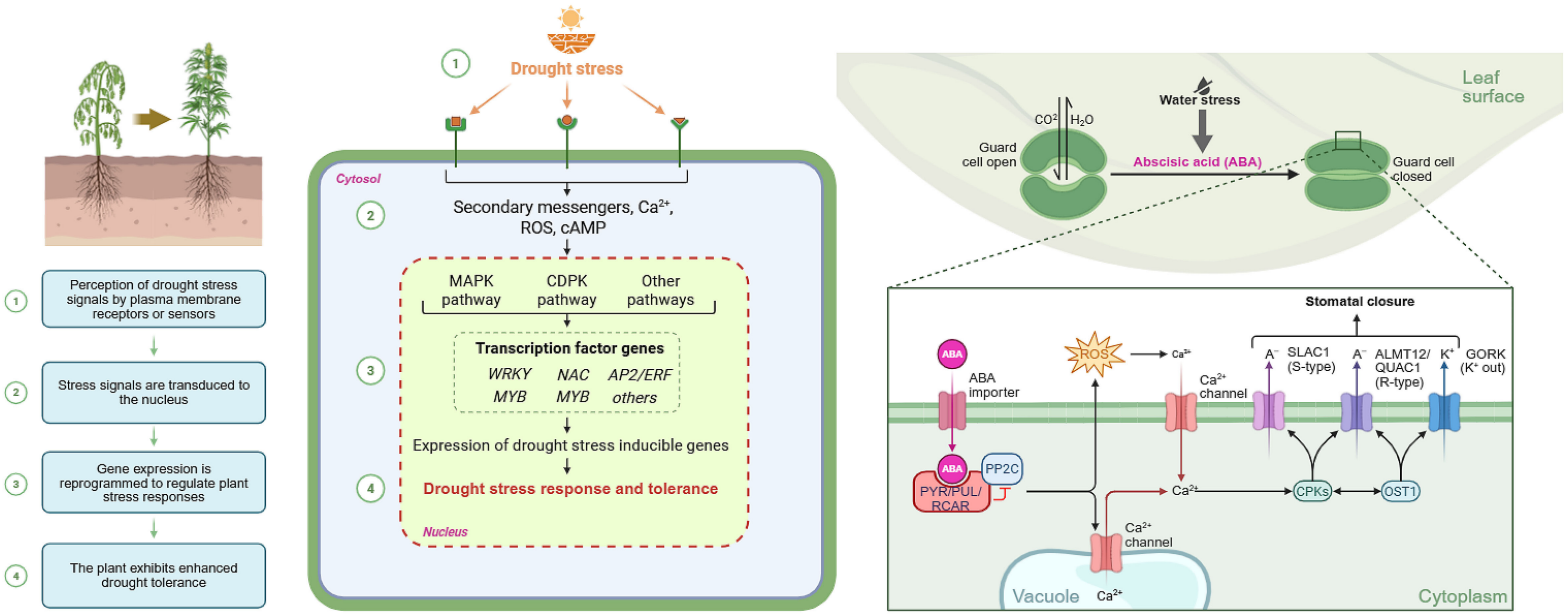
Overview of drought stress perception and signaling pathways in plants. Drought stress is perceived by membrane-associated sensors, leading to the production of secondary messengers, including Ca²^+^, ROS, and cAMP. These signals activate MAPK, CDPK, and other signaling pathways that regulate drought-responsive transcription factors and gene expression. Simultaneously, ABA signaling induces stomatal closure through activation of ion channels, thereby reducing water loss and enhancing drought tolerance. Image created by the authors using BioRender.com.

Stomatal aperture is regulated by an integrated signaling network involving abscisic acid (ABA), ion channel activity, reactive oxygen species (ROS), and hydraulic feedback mechanisms, which collectively modulate guard cell turgor under drought conditions (Cutler et al., 2010; Kim et al., 2010). Declining soil water availability reduces plant water potential, triggering ABA-mediated stomatal closure through drought-responsive signaling pathways (Zhu, 2016). Emerging transcriptomic evidence further supports the central role of ABA signaling in this process, as drought-stressed cannabis seedlings exhibit altered expression of ABA-responsive signaling components, including protein phosphatases (PP2C) and serine/threonine kinases (SnRK2), together with increased ABA accumulation, which has been associated with enhanced drought acclimation (Jiang et al., 2021). This response limits transportational water loss but also restricts CO_2_ diffusion, resulting in reduced photosynthetic activity and carbon assimilation (**Figure 1**) (Dimopoulos et al., 2025; Sharma et al., 2025).

Under drought stress, excess excitation energy in chloroplasts enhances ROS formation, which is counteracted by antioxidant defense systems including superoxide dismutase and catalase, maintaining cellular redox balance (Zhu, 2016). Consistent with these physiological adjustments, transcriptomic studies indicate that genes involved in chlorophyll biosynthesis, light-harvesting chlorophyll a/b-binding proteins (LHCs), and photosynthetic electron transport are differentially regulated under drought conditions, with many photosynthesis-related genes being downregulated, reflecting reduced chlorophyll content and a shift from energy capture toward protective energy conservation to minimize photodamage and ROS accumulation (Jiang et al., 2021). At moderate stress levels, however, partial stomatal closure may still optimize WUE while sustaining a degree of carbon gain (Eisele et al., 2016). In cannabis, photosynthetic efficiency may therefore be partially maintained under reduced stomatal conductance, reflecting tight coordination between gas exchange and carbon fixation, although this regulation is generally associated with reduced carbon gain and biomass production (Gill et al., 2025). Stable carbon isotope data further indicate improved intrinsic WUE under drought, supporting a conservative water-use strategy (Gill et al., 2022). Compared with several C3 crops, cannabis exhibits lower stomatal conductance at comparable photosynthetic rates, resulting in higher WUE than tomato, cotton, and grapevine, and values approaching those of sorghum, a crop recognized for high WUE (Singh and Reddy, 2014; Xue et al., 2021; Pazzagli et al., 2016; Lei et al., 2018; Tomás et al., 2012; Battle et al., 2024).

Drought can induce structural plasticity in stomatal traits, with stomatal size, density, and responsiveness to water deficit varying depending on genotype and stress intensity. Reduced leaf expansion combined with altered stomatal development may result in smaller but more densely distributed stomata. This plasticity represents an important adaptive strategy in cannabis, enabling regulation of transpirational water loss and improved tolerance to environmental stress (Haworth et al., 2024). However, contrasting evidence shows that stomatal density and size may remain unchanged under water deficit, suggesting that stomatal regulation can also occur predominantly through rapid aperture control rather than morphological adjustment (Gill et al., 2025; Lawson and Blatt, 2014). Together, these findings suggest that stomatal responses to drought in cannabis are likely genotype-dependent, spanning developmental plasticity and primarily physiological regulation. These traits enhance the speed and efficiency of stomatal regulation, improving responsiveness to fluctuations in water availability (McAusland et al., 2016; Westbrook and McAdam, 2021). Overall, this structural plasticity contributes to effective control of water loss under stress.

Beyond stomatal and structural regulation, biochemical adjustments further contribute to drought tolerance. Water deficit induces the accumulation of proline, a compatible solute associated with osmotic adjustment in plants (Hayat et al., 2012). In cannabis, drought stress has been associated with enhanced osmotic adjustment through increased proline accumulation, with levels rising more than twofold relative to the control (Gill et al., 2022). Elevated proline levels help maintain cellular turgor, stabilize proteins, and protect membranes under dehydration conditions (Raza et al., 2023). This osmotic adjustment enables cells to retain water and sustain metabolic activity despite reduced external water availability. Overall, proline-mediated biochemical regulation complements stomatal closure and structural adaptations, forming a multi-layered drought response strategy in cannabis.

In addition to proline accumulation, osmotic adjustment under drought may also involve dynamic changes in carbohydrate metabolism. Under drought conditions, soluble sugar and sucrose accumulation generally increase while starch reserves decline, reflecting carbon remobilization to maintain cellular osmotic balance. In cannabis, transcriptomic and physiological evidence further suggests that drought adaptation involves the regulation of starch and sucrose metabolism pathways, accompanied by altered expression of genes and enzyme activities associated with sucrose phosphate synthase (SPS), sucrose synthase, and invertase-related metabolism, highlighting the contribution of carbohydrate reprogramming to drought acclimation (Jiang et al., 2021).

At the whole-plant level, reduced stomatal conductance is integrated with hydraulic signaling driven by declining leaf water potential and increased xylem tension, reinforcing stomatal closure and conserving water. These responses are coordinated with adjustments in growth and cellular metabolism under water deficit conditions (Bista et al., 2018).

**Figure 2:**
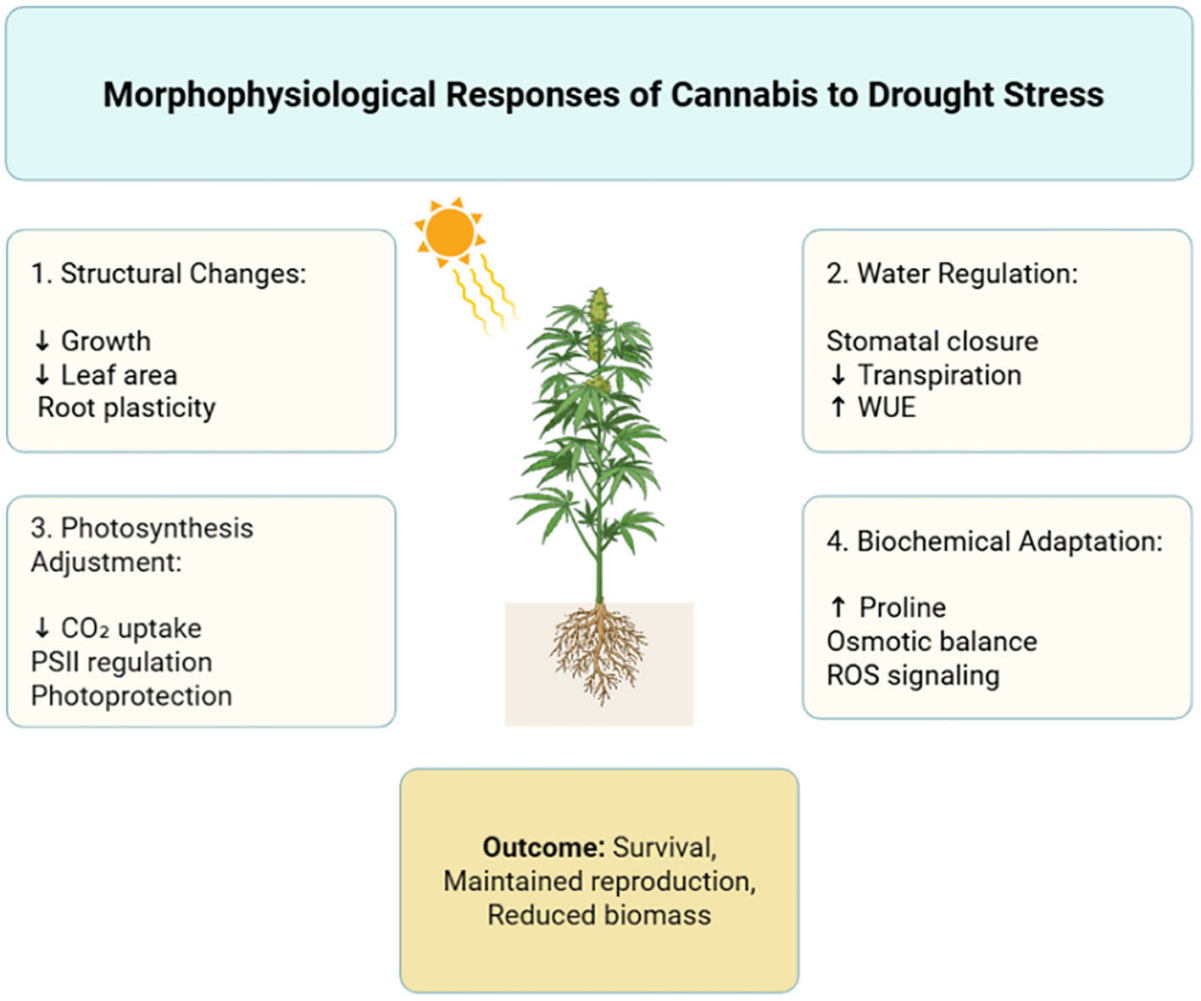
Schematic overview of the integrated morphophysiological responses of *C. sativa* to drought stress. Drought induces coordinated responses in *C. sativa*, including (1) structural adjustments, such as reduced shoot growth, decreased leaf area, and altered root system plasticity; (2) regulation of plant water status, characterized by stomatal closure, reduced transpiration, and improved WUE; (3) photosynthetic adjustments, including reduced CO_2_ assimilation, modulation of PSII activity, and activation of photoprotective mechanisms; and (4) biochemical adaptations, including proline accumulation, osmotic adjustment, and ROS signaling. Collectively, these responses enhance plant survival and support reproductive performance under drought stress, albeit at the expense of biomass accumulation. Abbreviations: WUE, Water-Use Efficiency; PSII, Photosystem II; ROS, Reactive Oxygen Species. Image created by the authors using BioRender.com.

A key feature of cannabis drought response is its high physiological plasticity and rapid recovery following rewatering (Gill et al., 2025). Stomatal conductance and photosynthetic activity recover quickly upon water availability, indicating that drought mainly imposes reversible functional constraints rather than permanent structural damage. This dynamic regulation of stomatal aperture enables flexible gas exchange control and enhances resilience under intermittent drought conditions (**Figure 2**).

### 3.3 Root system adaptations to drought stress

The root system is a fundamental vegetative organ responsible for anchorage, water and nutrient uptake, the synthesis and storage of organic compounds, and interactions with the soil environment. Owing to these roles, roots play a critical role in plant growth, adaptation to stress conditions, and crop yield (Siddiqui et al., 2021; Maqbool et al., 2022; Schneider et al., 2022; Özmen et al., 2023). Under drought conditions, root systems are central to maintaining water acquisition and supporting physiological processes that sustain plant growth and yield. However, phenotyping belowground root traits remain technically challenging in many plant species, including cannabis. Consequently, adaptive responses of the cannabis root system to drought stress are largely unexplored.

Root traits associated with drought tolerance can be classified into three main groups: (i) root architecture, primarily regulated by auxin signaling, where genes such as *DRO1* and auxin transport regulators control root angle and rooting depth; (ii) root biomass, largely governed by transcription factors (TFs) that influence lateral root formation, root length, and hormonal regulation; and (iii) root anatomy, shaped by TFs controlling xylem development and hydraulic conductivity, thereby improving water transport efficiency and overall drought tolerance (Zhang et al., 2025).

Root system architecture (RSA) exhibits plasticity in response to environmental conditions, playing a critical role in both stress adaptation and yield formation (Maqbool et al., 2022). RSA encompasses the morphological traits, topological structure, and spatial distribution of roots within the growth medium (De Dorlodot et al., 2007; Koevoets et al., 2016), and is shaped by the interaction of genetic and environmental factors. An optimal RSA enables plants to efficiently acquire soil water, thereby mitigating the adverse effects of drought and reducing yield losses (Wasson et al., 2012).

Evidence suggests that RSA parameters in cannabis vary with flowering type. Female plants exhibit substantially larger root systems, both in mass and surface area, than monoecious and male plants, whose roots are on average approximately half the size of those of females (McGrail et al., 2025). Increased root system size is associated with enhanced water and nutrient uptake, as well as greater carbon allocation to the soil (Kell, 2011). Consequently, the larger root systems observed in female cannabis plants may contribute to improved drought resilience by enhancing water acquisition and sustaining growth under water-limited conditions.

RSA variability is a complex quantitative trait governed by multiple biological processes. Accordingly, the optimization of RSA has been proposed as an important strategy for improving drought tolerance and crop productivity. However, the subterranean nature of root systems limits the investigation of RSA traits and the identification of their underlying genetic determinants. While numerous genes controlling RSA have been characterized in model and crop species (**Table 1**), their roles in cannabis and the adaptive modifications of its root architecture under drought conditions is not well understood. Insights gained from these studies may help guide future research aimed at elucidating the genetic basis of drought adaptation in cannabis. Recently, greenhouse phenotyping of 46 genetically diverse cannabis genotypes combined with image-based root architecture analysis identified cannabis homologs of RSA-associated genes previously reported in maize, rice, and Arabidopsis analyze (Morales et al., 2026). Among these, several homologs correspond to functionally characterized loci, including *ZmDRO1, ZmRt1, OsNRT1, OsDRO1, AtDro1, AtNRT2.1*, and *AtUTR7*, which are involved in root growth angle, nutrient transport, and root system development. These findings suggest a degree of conservation in RSA regulatory networks across plant species. However, functional validation of these candidate genes in cannabis is still required.

**Table 1:** Root traits, genes, and drought-resistance mechanisms reported in other plant species that may provide insights into drought tolerance in *C. sativa*.

Category	Gene/TF	Effect/function	Plant species	Reference
Root Architecture	*DRO1*	Regulates auxin distribution at the root tip, affects root bending, and promotes deep or shallow rooting	Rice	(Uga et al., 2011, 2013a)
	*DRO2*	Influences deep root formation	Rice	(Uga et al., 2013b)
	*DRO3*	Influences deep root formation	Rice	(Uga et al., 2015)
	*qSOR1*	Affects shallow rooting	Rice	(Kitomi et al., 2020; Uga et al., 2012)
	*ZmDRO1*	Increases vertical root angle and enhances yield in maize	Maize	(Feng et al., 2022)
	*EXOCYST70A3*	Modulates auxin signaling, alters root curvature, and adjusts rooting depth	Arabidopsis	(Ogura et al., 2019)
	*ZmCIPK15*	Steepens root angle and improves adaptation to stress conditions	Maize	(Schneider et al., 2022)
	*ZmCIPK3*	Regulates seminal root growth and confers drought resistance	Maize	(C. Li et al., 2023)
	*ZmRSA3.1/ZmRSA3.2*	Shapes auxin-related root angle and depth	Maize	(Ren et al., 2022)
Root Biomass	*ZmPTF1 (bHLH)*	Increases lateral root number, root length, and lateral root development; activates ABA pathway	Maize	(Li et al., 2019)
	*ZmbZIP4 (bZIP)*	Enhances ABA accumulation, lateral root number, and primary root length; regulates root development and stress-response genes	Maize	(Ma et al., 2018)
	*OsERF48 (AP2/ERF)*	Increases lateral root density and primary root length	Rice	(Jung et al., 2017)
	*RRS1 (R2R3 MYB)*	Affects root development via OsIAA3; knockout increases root growth	Rice	(Gao et al., 2023)
	*ARF7/ARF19*	Cooperate with LBD genes in lateral root formation; promote hydrotropic lateral root development	Arabidopsis	(Okushima et al., 2007; Orosa-Puente et al., 2018)
	*ZmVPP1*	Increases lateral root number and root dry weight	Maize	(Wang et al., 2016)
	*ZmTIP1*	Regulates root hair length	Maize	(X. Zhang et al., 2020)
	*OsABA8ox2*	Enhances ABA and auxin accumulation, increases root biomass	Rice	(Y. Zhang et al., 2020)
	*CKX1/CKX3*	Promote root system development and increase root biomass	Arabidopsis	(Werner et al., 2011)
	*LRD (KNAT3 homolog)*	Negatively regulates root growth under water stress	Wheat	(Placido et al., 2020)
	*SWEET17*	Critical for root development and drought tolerance	Arabidopsis	(Valifard et al., 2021)
	*SWEET11/SWEET12*	Increase sucrose transport and support root development	Rice	(Ren et al., 2022)
Root Anatomy	*XND1*	Reduces root hydraulic conductivity; plays a negative role in drought resistance	Arabidopsis	(Tang et al., 2018)
	*OsNAC5/OsNAC9/OsNAC10*	Enhance stele, cortex, and epidermis development; increase root diameter and drought resistance	Rice	(Jeong et al., 2013, 2010; Redillas et al., 2012)
	*OsERF71*	Increases root aerenchyma and vascular cell layers; enhances root thickness and drought resistance	Rice	(D.-K. Lee et al., 2016)

## 4 Cannabinoid biosynthesis under water-deficit stress

Water-deficit stress initially disrupts primary metabolism by reducing stomatal conductance, CO_2_ assimilation, and photosynthetic carbon fixation, thereby limiting biomass accumulation and altering carbon partitioning (Osakabe et al., 2014; Selmar and Kleinwächter, 2013a). To maintain cellular homeostasis, cannabis undergoes metabolic reprogramming involving carbohydrate and nitrogen metabolism, hormone signaling, and oxidative stress responses (Gao et al., 2018; Jiang et al., 2021). Transcriptomic and physiological studies have demonstrated that drought affects genes and pathways associated with photosynthesis, porphyrin and chlorophyll metabolism, starch and sucrose metabolism, carbon and nitrogen metabolism, and plant hormone signaling, highlighting the central role of primary metabolism in drought adaptation (Jiang et al., 2021). These metabolic adjustments not only support osmotic regulation and energy balance but also influence the availability of carbon skeletons, reducing power, and metabolic precursors required for the biosynthesis of secondary metabolites, thereby establishing a mechanistic link between primary and secondary metabolism under water-deficit conditions (Selmar and Kleinwächter, 2013a).

Under optimal cultivation conditions, cannabis is typically grown under well-watered conditions with adequate nutrient availability, temperatures of approximately 25–30 °C (up to 35 °C in some cultivars), relative humidity of approximately 75% during development stage and 55–60% during the vegetative and flowering stages, and sufficient light intensity (approximately 1500 μmol m^-^² s^-^¹ PPFD) to maximize photosynthetic performance (Chandra et al., 2008, 2011; Eichhorn Bilodeau et al., 2019). These conditions maintain active primary metabolism and efficient photosynthesis, ensuring an adequate supply of carbon assimilates and metabolic precursors for the biosynthesis and accumulation of secondary metabolites. Secondary metabolites are organic compounds that are not directly involved in primary growth and development but are essential for plant adaptation and survival under environmental stress. They function mainly in defense, stress tolerance, and ecological interactions, and their production is highly responsive to environmental conditions (Sharma et al., 2025).

**Figure 3:**
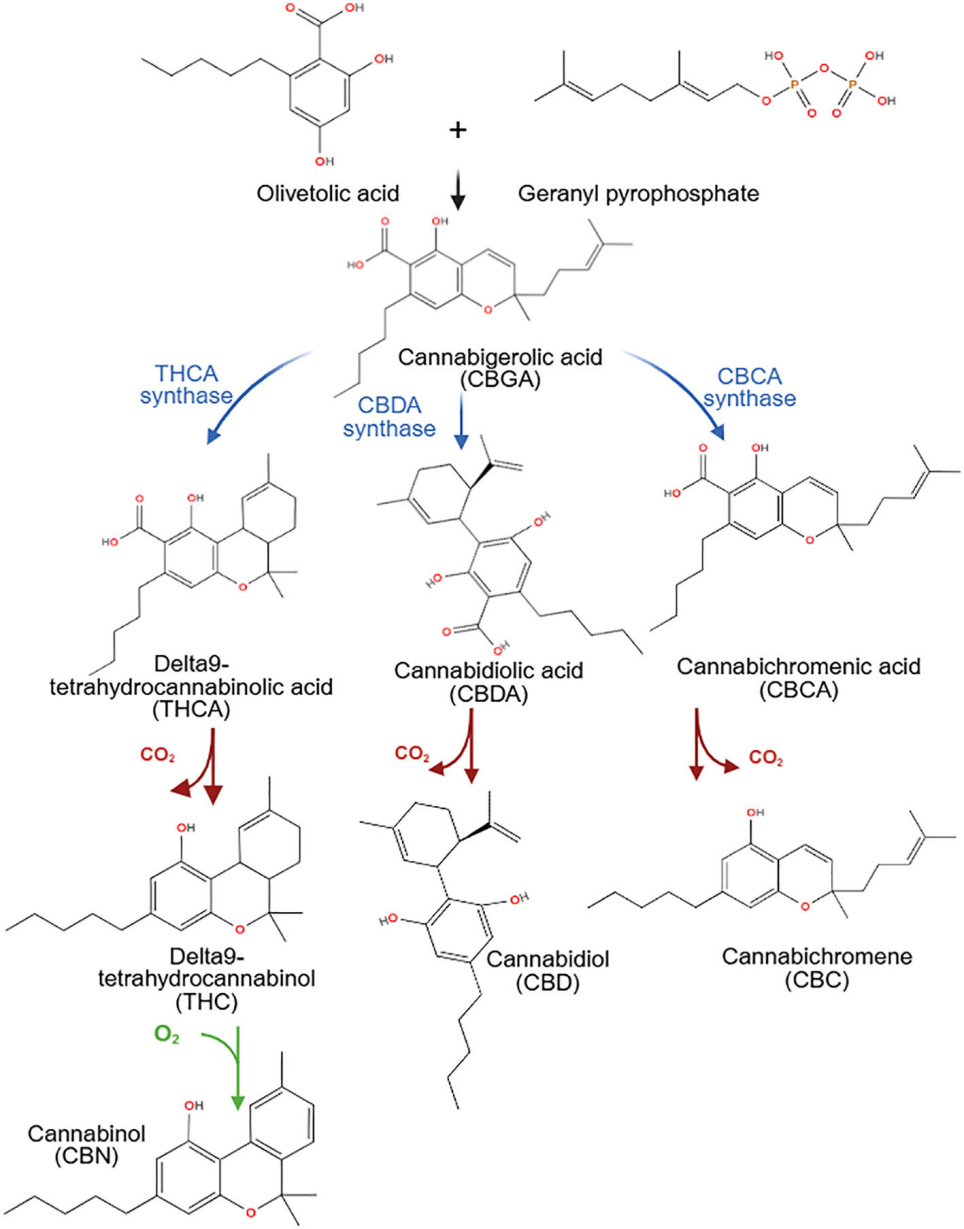
Biosynthetic pathway of major cannabinoids in *C. sativa*. Olivetolic acid, synthesized via the polyketide pathway, condenses with geranyl pyrophosphate (GPP), derived from the plastidial methylerythritol phosphate (MEP) pathway, to form cannabigerolic acid (CBGA), the central precursor of cannabinoid biosynthesis. CBGA is subsequently converted into Δ^9^-tetrahydrocannabinolic acid (THCA), cannabidiolic acid (CBDA), and cannabichromenic acid (CBCA) by THCA synthase, CBDA synthase, and CBCA synthase, respectively. Decarboxylation of these acidic cannabinoids releases CO_2_ and yields their neutral forms, Δ^9^-tetrahydrocannabinol (THC), cannabidiol (CBD), and cannabichromene (CBC). Under oxidative conditions during storage or aging, THC can be further converted to cannabinol (CBN). Image created by the authors using BioRender.com and molview.com.

In cannabis, the predominant classes of secondary metabolites include cannabinoids, terpenoids, flavonoids, and phenolic compounds (Jin et al., 2020). Among these, cannabinoids vary considerably in their composition. THC-type chemotypes typically contain high THC (0.5–15%) and low CBD (0.01–0.16%) content, whereas CBD-type chemotypes contain low THC (0.05–0.7%) and high CBD (1.0–13.6%) content (National Academies of Sciences, 2017). Cannabinoid biosynthesis is intrinsically linked to primary metabolism through the coordinated supply of metabolic precursors. Specifically, olivetolic acid is synthesized via the polyketide pathway, whereas geranyl diphosphate (GPP) is produced through the plastidial methylerythritol phosphate (MEP) pathway. The condensation of these two precursors generates cannabigerolic acid (CBGA), the central precursor for the biosynthesis of tetrahydrocannabinolic acid (THCA), cannabidiolic acid (CBDA), and cannabichromenic acid (CBCA) through the action of their respective oxidocyclase enzymes (**Figure 3**). Consequently, drought-induced alterations in primary metabolism may indirectly influence cannabinoid biosynthesis by affecting precursor availability and metabolic flux toward secondary metabolite production. Upon exposure to heat or prolonged storage, these acidic cannabinoids undergo decarboxylation to form their neutral counterparts, THC and CBD, whereas oxidative degradation of THC can result in the formation of cannabinol (CBN) (Thomas and ElSohly, 2016).

### 4.1 Glandular trichome responses to water-deficit stress

Glandular trichomes (GTs) are specialized epidermal secretory structures in cannabis and constitute the primary sites of cannabinoid and terpenoid biosynthesis and accumulation, particularly within stalked peltate trichomes of female inflorescences (Gonçalves et al., 2019; Dimopoulos et al., 2025). Understanding GT density and morphology is therefore critical for interpreting plant stress responses, as these structures serve as the primary sites for cannabinoid biosynthesis and storage (Alberti et al., 2025). In other GT bearing species, such as mints (*Mentha* spp.) and tomatoes (*Solanum lycopersicum*), various abiotic stresses, including water-deficit, have been shown to modify trichome activity or increase trichome density, suggesting a potential adaptive response that may also be relevant to cannabis (Turner et al., 2000; Khorasaninejad et al., 2011; Aryal et al., 2025). Preliminary observations and industry reports suggest that various types and intensities of abiotic or biotic stress may stimulate increased resin production or enhance the visual prominence of GTs in cannabis, potentially as part of an adaptive defense response. Such responses are biologically plausible, given that terpenes and cannabinoids can function as protective metabolites, helping mitigate oxidative damage, serving as antioxidants, or shielding developing reproductive tissues from environmental or biological threats. Nevertheless, the molecular mechanisms regulating trichome development in cannabis under drought or water-deficit stress are not yet fully resolved. In model plant systems, TFs from the MYB, bHLH, and WD−repeat (WD40) families act as key regulators of secondary metabolism and trichome initiation, often forming MYB-bHLH-WD40 (MBW) transcriptional complexes that control epidermal cell differentiation and specialized metabolite biosynthesis (Pattanaik et al., 2014).

### 4.2 Cannabinoid concentration vs total yield under water-deficit stress

Recent studies in cannabis indicate that moderate water−deficit stress can stimulate the accumulation of secondary metabolites, including elevated concentrations of cannabinoids. This suggests that controlled deficit irrigation may enhance THC and CBD levels by promoting stress−induced shifts in carbon allocation and secondary metabolism; however, any potential gains in phytochemical content must be weighed against the risk of biomass reduction and decreased overall yield under water−limited conditions (Sharma et al., 2025). A clear distinction must be made between cannabinoid concentration (expressed as a percentage of dry weight) and total cannabinoid yield (the absolute mass of cannabinoids produced per plant). Although moderate water−deficit stress can increase cannabinoid concentration, floral biomass is frequently reduced under water scarcity, meaning that total cannabinoid yield may remain unchanged or even decline despite higher concentrations. Under more severe drought conditions, both floral biomass and total cannabinoid production typically decrease, reflecting the overall negative impact of intense water stress on plant growth and secondary metabolite output (Cappello Fusaro et al., 2025). Unlike other horticultural crops, such as tomatoes or lettuce, where production systems are optimized to maintain ideal growing conditions that maximize fresh fruit yield or vegetative biomass, cannabis requires a more nuanced balance between growth optimization and controlled stress (Gonçalves et al., 2019; Park et al., 2022). While high−quality biomass production still depends on maintaining generally favorable environmental conditions, cannabinoid biosynthesis is often enhanced by exposing plants to carefully managed stressors, which can stimulate secondary metabolite pathways without compromising overall plant health and biomass (Addo et al., 2021; Ahsan et al., 2024). Thus, cannabis cultivation operates along a fine line between achieving maximum floral biomass and intentionally applying “less−than−ideal” conditions as a stimulus to promote secondary metabolite accumulation.

### 4.3 Hormonal and metabolic regulation of cannabinoid biosynthesis under water-deficit stress

Abscisic acid (ABA), jasmonic acid (JA), and ethylene exhibit extensive hormonal cross-talk during drought and water-deficit stress, integrating multiple signaling pathways. Collectively, these phytohormones coordinate physiological acclimation and metabolic reprogramming to enhance plant drought tolerance (Verma et al., 2016). By regulating stomatal closure and activating extensive stress-responsive gene expression networks, ABA functions as a central orchestrator of plant drought responses (Yoshida et al., 2014). In parallel, MeJA constitutes a critical regulator of defense-associated secondary metabolic pathways, particularly those governing the biosynthesis of terpenoid and phenylpropanoid derivatives (Wasternack and Hause, 2013; Howe et al., 2018). More specifically, in cannabis, exogenous methyl jasmonate (MeJA) has been shown to modulate GT development and alter the biosynthetic activity associated with cannabinoid production (Da Cunha Leme Filho et al., 2025a, b). In this sense, the application of MeJA can increase trichome density in cannabis; however, this increase is not necessarily correlated with higher cannabinoid levels (Da Cunha Leme Filho et al., 2026).

Exogenous MeJA acts through activation of the conserved jasmonate signaling cascade, resulting in downstream transcriptional reprogramming (Wasternack and Hause, 2013; Howe et al., 2018; Da Cunha Leme Filho et al., 2025a, b). Upon application, MeJA is converted to the bioactive jasmonoyl−isoleucine (JA−Ile), which binds to the SCF^COI1–JAZ coreceptor complex, promoting ubiquitin−mediated degradation of JAZ repressors and thereby releasing MYC−type TFs (e.g., MYC2, MYC3, and MYC4) (Kazan and Manners, 2013; Howe et al., 2018).

The released TFs activate jasmonate−responsive genes associated with secondary metabolism and epidermal differentiation, particularly those controlling GT initiation, cell proliferation, and secretory activity (Wasternack and Hause, 2013; Da Cunha Leme Filho et al., 2025a). Among these TFs, CsMYC4 has been implicated as an important regulator in cannabis, linking MeJA signaling to the development and density of capitate−stalked GTs, which are the primary sites of cannabinoid biosynthesis (Da Cunha Leme Filho et al., 2025a). In addition to CsMYC4, other transcriptional regulators, including MYB and HD−ZIP proteins (i.e. homeodomain-leucine zipper), are also involved in MeJA−induced transcriptional networks, where they regulate the expression of genes associated with cuticle formation, metabolite transport, and resin biosynthesis (Yan et al., 2017; Da Cunha Leme Filho et al., 2025b). MeJA enhances precursor availability, including geranyl diphosphate (GPP) and hexanoyl-CoA, thereby promoting olivetolic acid biosynthesis and increasing the biosynthetic capacity of GTs, which together improve upstream metabolic flux. This occurs despite the lack of consistent upregulation of terminal cannabinoid synthase genes such as THCAs or CBDAs and is accompanied by a redirection of carbon skeletons from vegetative growth toward defense-related metabolic pathways (Apicella et al., 2022; Da Cunha Leme Filho et al., 2025b).

**Figure 4:**
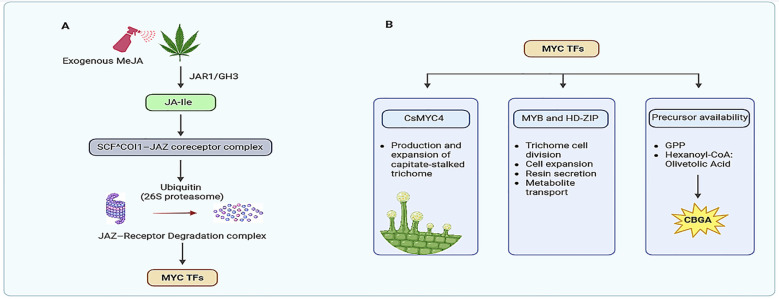
Proposed model of MeJA-mediated regulation of glandular trichome development and cannabinoid biosynthesis in *C. sativa*. **(A)** JA is converted to JA-Ile and perceived by the SCF^COI1–JAZ coreceptor complex, resulting in JAZ degradation and activation of MYC transcription factors. **(B)** MYC-mediated regulation of downstream targets, including CsMYC4, MYB, and HD-ZIP genes, leads to increased glandular trichome density, enhanced precursor flux, and promotion of cannabinoid biosynthetic capacity. Abbreviations: MeJA, Methyl Jasmonate; JA, Jasmonic Acid; JA-Ile, Jasmonoyl-Isoleucine; JAZ, Jasmonate ZIM-domain proteins; SCF^COI1, Skp1–Cullin–F-box CORONATINE INSENSITIVE1 complex; MYC, basic Helix–Loop–Helix transcription factor; HD-ZIP, Homeodomain-Leucine Zipper transcription factor. Image created by the authors using BioRender.com.

During floral development, MeJA responsive gene modules have been reported to show positive associations with GT density and may be related to cannabinoid accumulation, suggesting the existence of a coordinated regulatory relationship linking hormone signaling, trichome development, and secondary metabolite production (Da Cunha Leme Filho et al., 2025a, b). Further research is needed to clarify the relationship between MeJA application, GT density, and cannabinoid accumulation in cannabis. While a recent study had shown that exogenous MeJA application can increase trichome density, these structural changes were not consistently associated with corresponding increases in cannabinoid content (Da Cunha Leme Filho et al., 2026). The molecular mechanism underlying MeJA perception and signal transduction, including its effects on transcriptional regulation, trichome development, and secondary metabolite biosynthesis, is schematically illustrated in **Figure 4**.

### 4.4 Carbon allocation and oxidative signaling

ABA-induced stomatal closure, accompanied by the consequent reduction in internal CO_2_ availability, constitutes the primary mechanism by which water−deficit stress diminishes photosynthetic carbon assimilation (Osakabe et al., 2014). The growth-defense trade-off idea states that carbon resources may be diverted from biomass accumulation to secondary metabolism when growth is restricted under mild stress (Selmar and Kleinwächter, 2013a). Concurrently, drought-induced accumulation of ROS can activate redox-sensitive TFs that upregulate the production of protective secondary metabolites, including phenolic and terpenoid compounds (Nakabayashi and Saito, 2015). This underscores a central challenge in cannabis cultivation: optimizing production systems to sustain robust biomass accumulation while simultaneously eliciting the physiological cues necessary to enhance secondary metabolite synthesis.

Cannabinoids exhibited an antioxidant activity *in vitro*, with compounds such as Δ^9^-THC and CBD shown to effectively scavenge ROS and prevent oxidative damage in cellular and chemical systems (Russo, 2011; Watt and Karl, 2017). Given that drought stress is associated with increased oxidative pressure and metabolic reprogramming in plants, changes in cannabinoid accumulation under water-deficit conditions may reflect a stress-induced shift in secondary metabolism. Increases in THC under mild water-deficit conditions may represent an adaptive response associated with oxidative stress mitigation or protection of reproductive tissues; however, direct in planta evidence supporting this functional role in cannabis is limited. This ultimately constrains biomass formation and secondary metabolite biosynthesis, thereby reducing overall cannabis yield per plant (Selmar and Kleinwächter, 2013b). Taken together, these observations suggest a biphasic relationship between drought severity and cannabinoid biosynthesis, in which moderate water−deficit stress may enhance cannabinoid concentrations, whereas more severe or prolonged stress suppresses metabolic activity and ultimately reduces overall cannabinoid production.

## 5 RNA-mediated and epigenetic regulation of drought responses

Water deficit leads to reduced cell turgor, impaired photosynthesis, oxidative damage and altered nutrient transport. To survive these conditions, plants rapidly reprogram gene expression through complex regulatory networks that operate at transcriptional, post transcriptional and epigenetic levels (Gelaw and Sanan-Mishra, 2021). Drought perception triggers signaling cascades involving calcium ions, ROS and phytohormones particularly ABA. These signals activate drought responsive TFs such as DREB, NAC, MYB and bZIP, which regulate the expression of stress responsive genes (Gao et al., 2018; Ali et al., 2025). These genes encode proteins involved in osmotic adjustment, antioxidant defense, stomatal regulation and protective molecules like late embryogenesis abundant (LEA) proteins and dehydrins (Ali et al., 2025). In addition to transcriptional regulation, RNA-mediated mechanisms play an essential role in fine tuning drought responses (Bolc et al., 2025). Drought-induced molecular regulatory networks have been widely studied in model and crop species; however, such mechanisms remain comparatively underexplored in cannabis. Recent transcriptomic studies in cannabis have nonetheless begun to uncover drought-responsive regulatory networks, identifying more than 1200 differentially expressed genes associated with hormone signaling, photosynthesis, oxidative stress, carbohydrate metabolism, and stress-related transcription factors, indicating that drought adaptation in cannabis involves extensive molecular reprogramming (Gao et al., 2018). Considering the high degree of conservation of stress-responsive pathways across angiosperms, insights from other plant systems provide a valuable framework for understanding potential regulatory mechanisms in cannabis.

### 5.1 Non-coding RNAs in drought stress regulation

**Figure 5:**
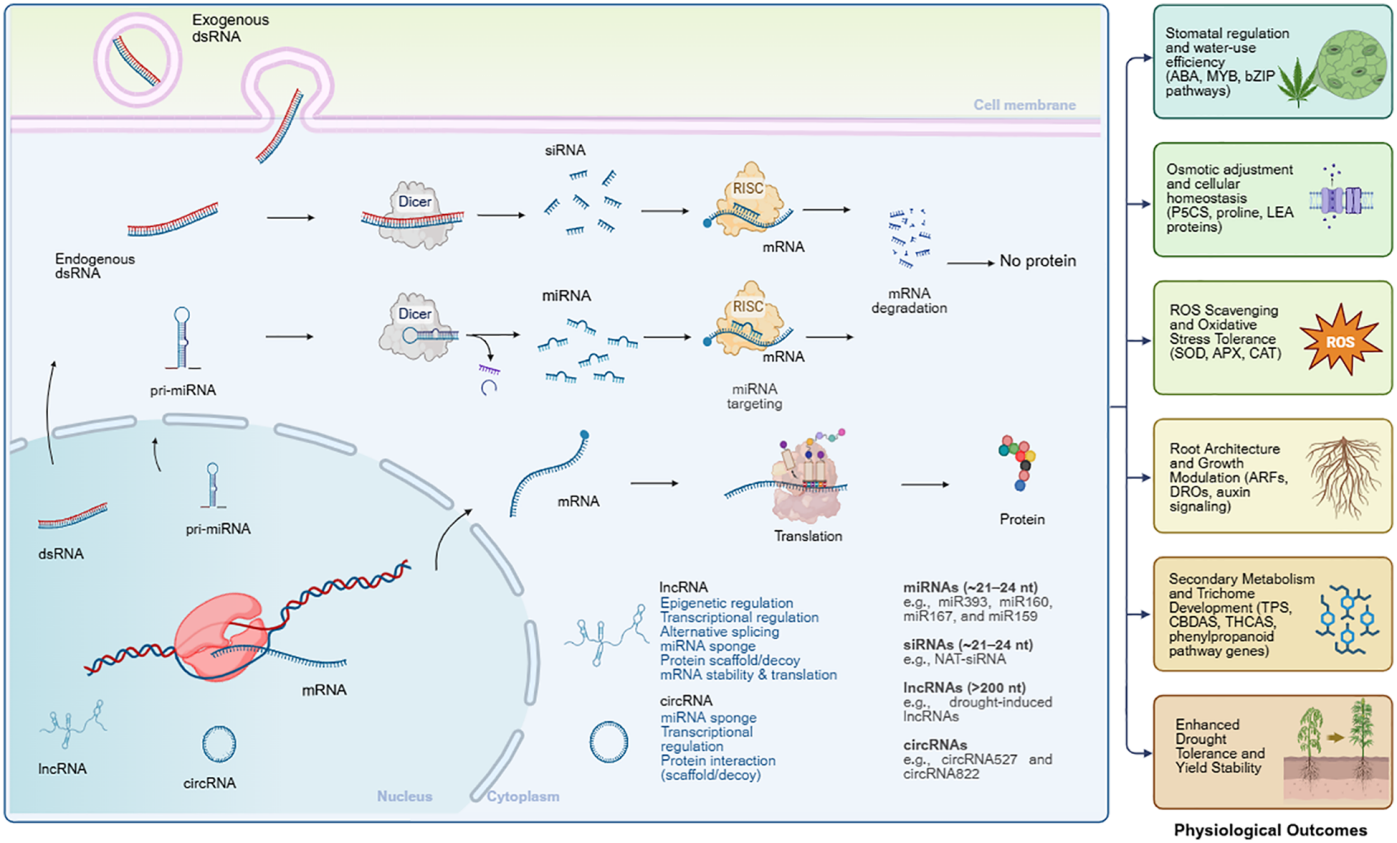
Schematic representation of the biogenesis and regulatory functions of plant non-coding RNAs involved in drought stress responses. Endogenous and exogenous dsRNA are processed by Dicer into siRNAs, whereas pri-miRNAs give rise to mature miRNAs. Both siRNAs and miRNAs are loaded into the RNA-induced silencing complex (RISC) to direct mRNA cleavage or translational repression. In parallel, lncRNAs and circRNAs modulate drought-responsive gene expression through epigenetic, transcriptional, and post-transcriptional regulatory mechanisms. Image created by the authors using BioRender.com.

Non-coding RNAs (ncRNAs) act as central regulators of plant drought tolerance by controlling gene expression, hormone signaling, chromatin modification, and stress-responsive pathways (**Figure 5**) (Bolc et al., 2025). MicroRNAs (miRNAs) represent the most extensively studied class of ncRNAs and play a pivotal role in post-transcriptional gene regulation under drought stress. Drought-responsive miRNAs display tissue-, stage- and genotype-specific dynamics, with many families conserved across angiosperms but exhibiting species-dependent changes (Bolc et al., 2025). These small RNAs generally contribute to the trade-off between growth and survival by modulating the auxin and ABA pathways, root architecture, leaf polarity/hydraulics, and oxidative stress responses (Zhang et al., 2022). The miR393 module modulates drought adaptation by targeting auxin F-box receptors such as TIR1 and AFB proteins, thereby suppressing auxin perception during water deficit (Xia et al., 2012; Fard et al., 2017). The miR160-ARF10/16/17 and miR167-ARF modules regulate auxin-ABA cross talk and root architecture, optimizing root system plasticity and adaptation to soil moisture limitation. These miRNAs fine tune auxin signaling to balance root growth and drought survival (Tang and Chu, 2022; Zhang et al., 2022). The miR159-MYB pathway fine tunes ABA sensitivity during drought, miR159 regulates MYB TFs (*MYB33/101/65*) coordinating seedling growth and ABA-mediated stress responses under water deficit. miR398 regulates Cu/Zn superoxide dismutase genes that control ROS homeostasis. These miRNA-mediated regulatory modules contribute to modulation of ABA signaling and stress-responsive gene expression under water deficit conditions. Similarly, miR156 modulates SPL TFs affecting root architecture and developmental plasticity under water deficit. These miRNA-target interactions coordinate physiological responses including stomatal regulation, osmotic balance, and antioxidant activity during drought stress (Zhakypbek et al., 2025).

In cannabis, targeted regulatory relationship between miRNA and mRNA under salt stress identified differentially expressed genes mainly enriched in plant hormone signal transduction, the MAPK signaling pathway, and starch and sucrose metabolism. The network also identified 230 miRNA–mRNA interactions involving 16 miRNAs, with significant regulation of the highly conserved miR156 family. miRNA156 expression increases salt stress tolerance and helps the plant withstand stress conditions until conditions become suitable. The plant hormone signal transduction pathway revealed that the key hub genes were related to proteins TIFY 6B and phosphatase 2C8. It was also speculated that interplay between TIFY family-related genes and PP2C-related genes inhibited ABA signaling and promoted jasmonate signal transduction (Cao et al., 2023; Kasprowiak et al., 2025).

Another study on the expression of MYB transcription factor family during cannabis seed germination under salt stress identified the transient expression of *MYB33* and *MYB44* during the initial osmotic phase, while the sustained expression of *MYB14*, *MYB78*, and *MYB79* in the prolonged adaptation phase. These TFs were predicted to interact with proteins participating in crucial stress-response pathways, including components of ABA signaling (e.g., protein phosphatases 2C and SnRK2 kinases) and enzymes accountable for ROS homeostasis (e.g., peroxidases and catalases). Extensive cross-talk among different MYB TF family members themselves, as well as with other families of stress-related TFs (e.g., bZIPs, NACs), was also predicted suggesting coordinated multi-level transcriptional regulation (Wang et al., 2026). The significant enrichment of ABA-Responsive Elements (ABREs) in the promoters of key MYBs, such as *MYB14/78/79*, aligns their predicted regulatory activity with the core ABA signaling pathway, a central regulator of seed germination and abiotic stress responses (Seo et al., 2009; C. Li et al., 2015). Building on the regulatory complexity of MYB TF-mediated stress responses, transcriptomic profiling of drought-stressed cannabis has revealed differential regulation of multiple TF families, including NAC, MYB, WRKY, AP2/ERF, and bHLH members (Gao et al., 2018), with NAC TFs being consistently upregulated, suggesting their contribution to drought-responsive transcriptional reprogramming in cannabis.

In another genome-wide study of the ascorbate peroxidase (APX) gene family in cannabis under drought, cold, salt, and oxidative stress, *CsAPX2* showed notably higher expression under drought conditions compared to other stress treatments (Liang et al., 2024). During drought exposure, *CsAPX4*, *CsAPX5*, and *CsAPX8* were initially downregulated within the first 6 h, whereas other *CsAPX* genes exhibited increased expression. *CsAPX3* and *CsAPX6* showed similar temporal patterns, with peak expression at 12 h, while *CsAPX1*, *CsAPX5*, and *CsAPX8* reached maximum expression at 48 h, indicating dynamic and gene-specific regulation over time. Notably, *CsAPX7* responded rapidly, peaking at 6 h, whereas *CsAPX2* displayed the strongest upregulation (approximately 22-fold) at 12 h. These findings suggest that members of the *CsAPX* gene family may play important roles in oxidative stress regulation and drought response in cannabis (Liang et al., 2024).

In addition to TF regulation, miRNAs also influence physiological responses associated with drought tolerance, including stomatal closure, osmotic adjustment, root architecture modification, and antioxidant defense (Wang et al., 2020; Zhang et al., 2022). These processes are mediated through interactions with key hormonal and stress-signaling pathways. Recent studies further demonstrate that miRNAs function within complex regulatory networks involving feedback loops with TFs and interactions with long non-coding RNAs acting as target mimics. These multilayered regulatory circuits enable precise tuning of gene expression and facilitate adaptive responses to prolonged drought conditions (Lei et al., 2025).

Beyond miRNA-mediated pathways, other classes of ncRNAs, particularly long non-coding RNAs (lncRNAs), contribute to the regulation of drought stress responses. lncRNAs have been implicated in modulating various abiotic stresses in response to drought, salinity, temperature, hypoxia, and others (Kumar Saroha et al., 2026). lncRNAs participating in drought-responsive regulation have been studied in maize (Zhang et al., 2014), cotton (Lu et al., 2016), Arabidopsis (Qin et al., 2017), cassava (Li et al., 2017), wheat (Cagirici et al., 2017), and cultivated rice (Yuan et al., 2018), implying that lncRNAs as ubiquitous regulators are involved in responding to drought stress in various kinds of plant species. lncRNAs function in both cis and trans to regulate transcription, act as miRNA decoys, and coordinate hormone signaling redox balance, and developmental responses, while circular RNAs (circRNAs) act as miRNA sponges and modulate stress-related genes such as *DREB*, *RD29*, and *RPS5*, improving drought tolerance (Bao et al., 2025).

### 5.2 Epigenetic regulation and drought memory

**Figure 6:**
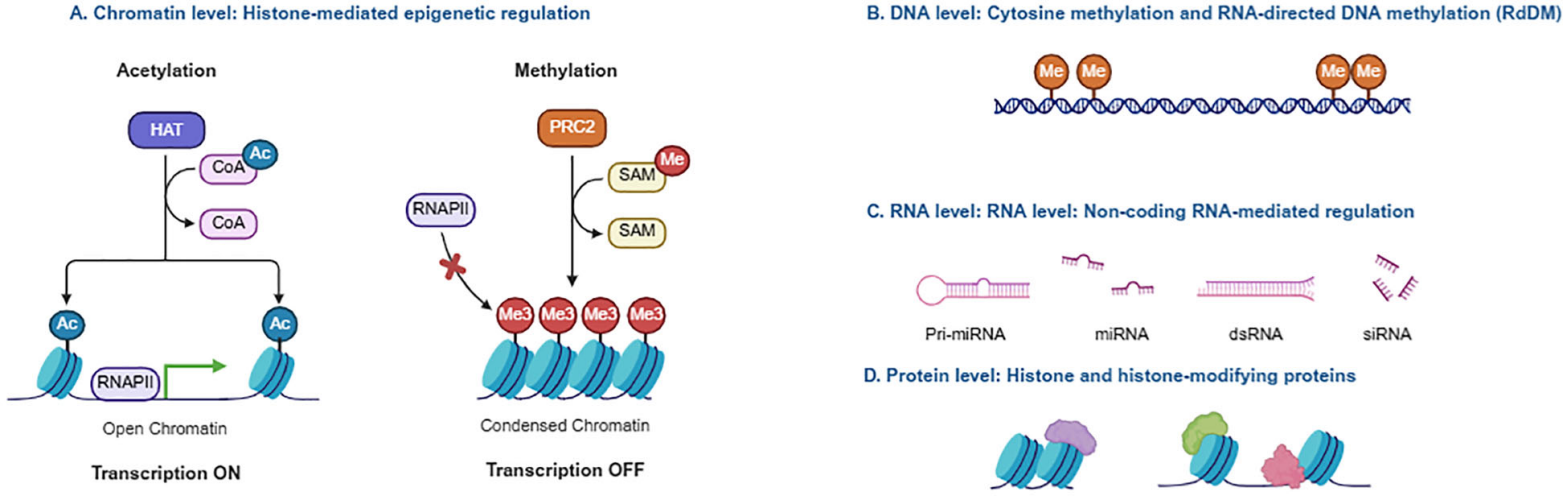
Schematic overview of epigenetic mechanisms regulating plant drought responses. **(A)** Histone modifications, **(B)** DNA methylation and RNA-directed DNA methylation (RdDM), **(C)** non-coding RNAs, and **(D)** chromatin-associated proteins coordinately regulate chromatin organization and gene expression, thereby contributing to plant adaptation to drought stress. Image created by the authors using BioRender.com.

Epigenetic regulation plays a central role in plant responses to drought stress by enabling heritable yet reversible changes in gene expression without altering the DNA sequence. In plants, DNA methylation, histone modifications, and RNA-mediated processes constitute a fundamental regulatory layer controlling stress-responsive gene expression (**Figure 6**). However, epigenetic regulation of drought responses in cannabis is not yet well understood. Given the conserved nature of these mechanisms across plant species, similar regulatory processes are likely to operate in cannabis, although further investigation is required.

Drought stress induces dynamic changes in DNA methylation patterns, leading to activation or repression of stress-related genes while maintaining genome stability through transposon silencing (Rehman et al., 2022; Ramakrishnan et al., 2022; Liu et al., 2023; Ding et al., 2024). Genome-wide bisulfite sequencing studies in crops such as maize, wheat, barley, and cotton reveal locus-specific hypomethylation associated with genes involved in osmolyte biosynthesis, ROS scavenging, and ABA signaling (Chwialkowska et al., 2016; Zhu, 2016; Wang et al., 2021; Naderi et al., 2024). Importantly, some methylation marks persist after stress removal, forming the basis of epigenetic memory that enables plants to responds more efficiently to subsequent drought events (Ding et al., 2024; Ma et al., 2024). These findings suggest that comparable methylation dynamics may contribute to drought-induced transcriptional regulation in cannabis, although direct evidence is currently lacking.

Histone modification further refines drought-responsive gene regulation through post-translational changes such as acetylation and methylation. Histone acetylation marks such as H3K9ac and H3K27ac are associated with transcriptional activation by loosening chromatin structure, whereas histone methylation marks exhibit context dependent effects, with H3K3me3 correlating with active transcription and H3K27me3 associated with gene repression. ABA plays an important role during plant growth and development. When exposed to drought stress, plants exhibit increased H3K4me3 modification, which is associated with ABA synthesis and is involved in enhanced regulation of *NCED3* (*ninecisepoxycarotenoid dioxygenase 3*) gene (Junaid et al., 2024). *NCED3* regulates ABA biosynthesis during drought (Sato et al., 2018). *Brassica napus* exhibits H3K4me3 gain and H3K27me3 loss at proline synthesis loci, indicating a role in drought tolerance (Prasad et al., 2025). In maize, circRNAs in roots are associated with H3K36me3 and H3K4me1 marks, suggesting a role in regulating drought response (Xu et al., 2024). During drought stress, stress-inducible genes often acquire activating histone marks, while growth-related genes are repressed, enabling resource reallocation toward survival. ROS accumulation is another indicator for plants to activate their response against drought stress, and is involved on ABA signaling pathways, Ca^2+^ flux and sensing water scarce conditions. ROS-related plant response and ROS-dependent DNA methylation is linked with regulation of ABA gene expression (Kim et al., 2019). High ROS leads to small RNA modifications and their unbalanced interaction with proteins leading to apoptosis (Dumont and Rivoal, 2019). Notably, certain histone marks persist at stress responsive loci even after the cessation of stress, maintaining these genes in a transcriptionally competent or poised state, a hallmark of transcriptional priming that underlies stress memory. These mechanisms are likely conserved in cannabis and may play a role in coordinating ABA signaling and stress adaptation under water deficit.

Chromatin remodeling complex including SWI/SNF, ISWI and CHD families, reposition nucleosomes in an ATP-dependent manner, thereby facilitating or restricting access of TFs to DNA. These complexes are essential for rapid transcriptional reactivation during recurrent drought stress, as they help maintain chromatin in a semi-open configuration at previously induced loci (Huang et al., 2025). In parallel, RNA-mediated pathways particularly, RNA directed DNA methylation (RdRM) integrate small interfering RNAs (siRNAs) with epigenetic modifications by guiding *de novo* methylation to specific genomic regions via AGO proteins and plant specific RNA polymerases (Pol IV and Pol V) (Zhang et al., 2012). This mechanism reinforces transcriptional silencing of transposable elements and modulates stress-responsive genes contributing to both genomic stability and long-term regulatory memory.

Epigenetic memory in plants allows stress-induced changes in gene expression to persist without altering the DNA sequence, helping plants respond more effectively to recurring abiotic stresses. Increasing evidences prove the role of stress-induced epigenetic memory in the form of epi-alleles being transmitted across generations (Molinier et al., 2006). This memory not only helps the plants to respond to the stress instantly, as well as modifies adaptive behaviors in the offspring (Ashapkin et al., 2020). Epigenetic memory in plants is primarily mediated by DNA methylation and histone modifications (Liu and He, 2020) through the regulation of key genes such as *SOS1*, *RD29A* and *ONSEN* in response to various stresses (Kang et al., 2022), enabling the transgenerational inheritance of epigenetic marks and thereby contributing to plant adaptability and evolution (Ahtisham and Obaid, 2025).

Plants memorize stress most likely through epigenetic processes in the germ line and transmit it across generations through mitotic or meiotic divisions (Lang-Mladek et al., 2010). The established somatic memory across mitotic divisions can also be transmitted meiotically to progeny. Epigenetic regulation involves both DNA methylation and histone modifications, which together contribute to stress memory and adaptive responses. In rice under heavy metal stress, DNA methylation has been shown to mediate transgenerational memory, leading to the activation of heavy metal-transporting P-type ATPase genes (HMAs) after stress removal (Cong et al., 2019). In *B. napus*, drought stress triggered extensive DNA methylation reprogramming, with contrasting patterns between sensitive and tolerant genotypes (Huang et al., 2026). Additionally, histone deacetylation mechanisms, such as those mediated by MdHDA6, have been shown to regulate drought-responsive genes and negatively affect drought resistance in apple (W. Li et al., 2023).

Genome-wide studies in model and crop species such as maize, rice, wheat, and Arabidopsis have identified hundreds to thousands of drought-responsive ncRNAs associated with ABA signaling, ROS detoxification, osmoprotection, root system remodeling, and water-use efficiency. However, comparable evidence in cannabis is limited and largely uncharacterized. Functional studies, including circ032768-miR472-RPS5 and the lncRNA DRIR module, further illustrate the direct contribution of ncRNAs to enhanced drought tolerance. Collectively, these findings support a model in which ncRNAs operate as a multilayered regulatory network controlling water uptake and transport, stress signaling, and metabolic adjustment. Despite their considerable potential as targets for breeding and genome editing, the majority of ncRNAs still lack functional validation, particularly in cannabis.

## 6 Microbial modulation of drought responses in cannabis

Plant growth-promoting microbes in the rhizosphere, especially plant growth−promoting rhizobacteria/fungi (PGPR/PGPF) and arbuscular mycorrhizal fungi (AMF), can substantially enhance the drought tolerance of cannabis by mediating plant-microbe interactions in the root zone (Dong et al., 2019). Although exogenous microbial inoculation of cannabis under drought has been studied far less than its potential would suggest, several recent reports highlight promising strains.

Yuan et al. (2024) demonstrated that inoculation with the AMF *Funneliformis mosseae* alleviated severe drought stress in cannabis. Under an extreme drought regime of 15% field capacity, *F. mosseae* increased chlorophyll content by 26.3% and relative water content (RWC) by 15.1% compared with non−inoculated controls. Yield-related parameters also improved substantially, with fresh biomass reaching 110.4% and dry biomass 125.0% of the control levels. The observed reduction in malondialdehyde (MDA) content suggests attenuation of oxidative damage, while enhanced proline accumulation indicates improved osmotic adjustment and water-use regulation under drought.

**Figure 7:**
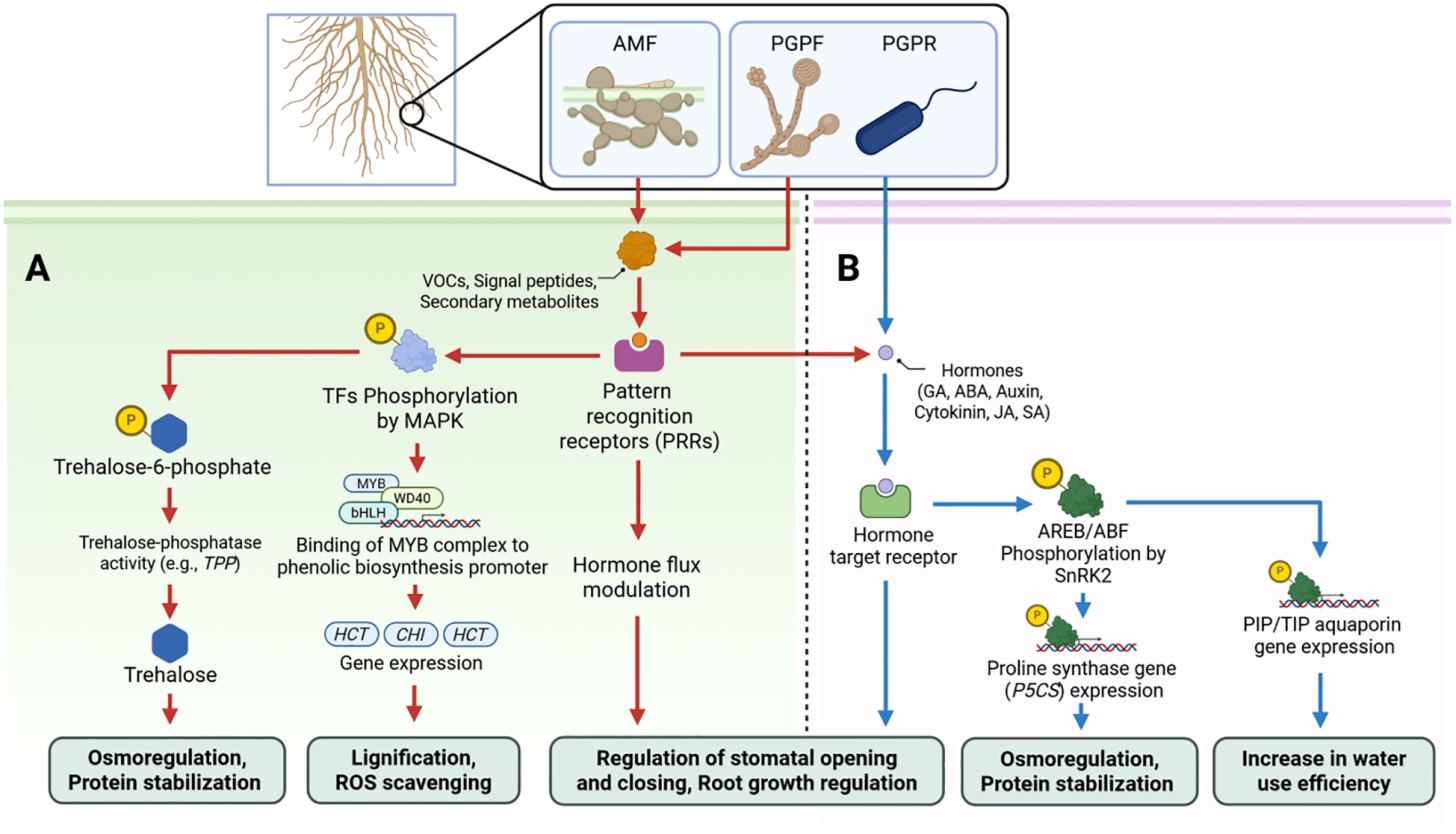
Schematic diagram of beneficial microbe-mediated drought mitigation mechanisms observed in *C. sativa*. **(A)** Microbial metabolite-mediated drought mitigation. **(B)** Phytohormone-mediated drought mitigation. PGPR, Plant Growth-Promoting Rhizobacteria; PGPF, Plant Growth-Promoting Fungi; AMF, Arbuscular Mycorrhizal Fungi; GA, Gibberellic Acid; ABA, Abscisic Acid; JA, Jasmonic Acid; SA, Salicylic Acid; VOCs, Volatile Organic Compounds. Image created by the authors using BioRender.com.

Similarly, application of the PGPF *Trichoderma hamatum* to cannabis subjected to a 10-day irrigation suspension resulted in 304.3% increase in photosynthetic rate and a 275.6% increase in WUE relative to drought-stressed controls. Inoculated plants also showed higher chlorophyll a and b contents (28.9% and 38.6%, respectively), indicating stabilization of the photosynthetic apparatus and maintenance of productivity under water−limited conditions (Ryu et al., 2025). Collectively, these findings underscore the substantial promise of beneficial rhizosphere microorganisms as a strategy to mitigate drought stress and sustain productivity in cannabis (**Figure 7**).

### 6.1 Microbe−induced secondary metabolism under drought

Beneficial microorganisms, including PGPR and AMF, can reprogram the metabolic networks of cannabis to promote the synthesis of diverse secondary metabolites that enhance cellular survival and maintain homeostasis under extreme drought conditions. In this context, these microbes function not only as facilitators of nutrient acquisition but also as biostimulants that activate plant immune signaling and metabolic regulatory pathways (Pagnani et al., 2018).

Evidence from *A. thaliana* demonstrates that microbial inoculants and microbe−derived elicitors can activate induced systemic resistance (ISR) while priming the phenylpropanoid and flavonoid pathways (Mathys et al., 2012; Ali and McNear, 2014; Liu et al., 2019). Plants in an ISR-primed state exhibit more rapid and robust activation of secondary metabolism-related genes upon exposure to stress, resulting in greater accumulation of protective metabolites compared with non−primed plants (Mathys et al., 2012). This enhanced responsiveness has been associated with endophytic and rhizosphere microorganisms that elevate basal expression levels of TFs governing secondary metabolism such as MYB, bHLH, WRKY and induce epigenetic modifications in the promoters of key phenylpropanoid genes. Together, these changes increase the plant’s capacity to respond efficiently to subsequent environmental stressors (Alwutayd et al., 2023) (**Figure 7A**).

Ryu et al. (2025) further demonstrated that *T. hamatum*, isolated from open−field cannabis soils, enhanced both cannabinoid accumulation and antioxidant secondary metabolites in drought-stressed cannabis. Relative to drought-stressed controls, inoculated plants exhibited a 27.95% increase in total phenolic content (TPC) and a 29.10% increase in total flavonoid content (TFC). These increases were tentatively associated with the upregulation of chalcone isomerase (CHI) and shikimate O−hydroxycinnamoyl transferase (HCT), key enzymes in the flavonoid and phenylpropanoid biosynthetic pathways (**Figure 7A**). Notably, the combined application of drought stress and *T. hamatum* resulted in higher TPC and TFC levels than either treatment alone. In the absence of substantial changes in enzymatic antioxidant activities, the authors proposed that *T. hamatum* enhances drought tolerance primarily through stimulation of non-enzymatic antioxidant systems (Ryu et al., 2025).

In addition, increased expression of lignin biosynthesis-related genes, including *PAL*, *C4H*, *4CL*, and *CAD*, contributes to enhanced lignification, thereby reducing transpirational water loss and reinforcing cell walls against drought−induced structural damage (Ramaroson et al., 2022). In cannabis, the *T. hamatum*−induced overexpression of *HCT* is particularly notable, as *HCT* serves as a central regulatory node in lignin biosynthesis (Chao et al., 2021) (**Figure 7A**). These findings parallel those observed in model plant systems and further emphasize the role of microbially mediated secondary metabolism in conferring drought resilience in cannabis.

Under drought conditions, a critical physiological priority for plant cells is the reduction of cellular water potential to sustain water uptake from desiccated soils and maintain turgor, thereby preserving tissue integrity (Mikiciuk et al., 2024). Beneficial root-associated microorganisms can facilitate this process by promoting the accumulation of compatible solutes, including proline, glycine betaine, soluble carbohydrates, and trehalose (Gasemi et al., 2023) (**Figure 7A**).

Certain AMF, including *Rhizophagus irregularis*, contribute to enhanced drought tolerance by maintaining photosynthetic efficiency and modulating host carbon metabolism. These effects are associated with increased accumulation of soluble sugars, such as glucose and fructose, in colonized tissues (Gasemi et al., 2023). Such carbohydrates function not only as metabolic substrates but also as osmoprotectants that support cellular homeostasis under water-deficit conditions. Trehalose, a microbially synthesized disaccharide produced by AMF and other symbionts, including rhizobia, plays a pivotal role in plant desiccation tolerance. Trehalose stabilizes cellular membranes and proteins by promoting the formation of a vitrified, glass-like state during dehydration, thereby preserving structural integrity under severe water loss (Sharma et al., 2020). In cannabis, gene ontology (GO) enrichment analysis revealed that *T. hamatum* treatment significantly enriches pathways associated with trehalose biosynthesis and trehalose phosphatase activity (Ryu et al., 2025). These findings suggest that microbial inoculation activates an integrated stress-adaptive mechanism by stimulating the synthesis of compatible solutes, including trehalose and sucrose. Such metabolic reprogramming likely enhances intracellular water retention and mitigates protein denaturation during dehydration stress.

### 6.2 Microbial regulation of phytohormones in drought stress mitigation

Plant hormones are key signaling molecules that perceive environmental fluctuations and coordinate integrated physiological responses at the whole-plant level. Beneficial rhizosphere microorganisms can modulate phytohormonal pathways, thereby enhancing drought tolerance in host plants (Mathur and Roy, 2021).

ABA plays a pivotal role in drought adaptation. Under water-deficit conditions, ABA is synthesized primarily in roots and transported to aerial tissues, where it induces stomatal closure and reduces transpirational water loss (Daszkowska-Golec, 2016). At the molecular level, drought−induced ABA binds to PYL receptors, leading to inhibition of the negative regulator protein phosphatase 2C (PP2C). This inhibition releases SNF1-related protein kinases 2 (SnRK2), which subsequently activate TFs such as AREB/ABF. These TFs upregulate genes encoding ion channels required for stomatal closure, as well as genes involved in osmoprotectant biosynthesis and stress adaptation (Alwutayd et al., 2023) (**Figure 7B**).

Certain soil bacteria, including *Bacillus pumilus*, possess biosynthetic pathways for phytohormones such as gibberellic acid (GA) and ABA (Shaffique et al., 2024). Moreover, several PGPR genera, including *Bacillus* and *Azospirillum*, are known to synthesize and secrete ABA into the rhizosphere, directly contributing to the host plant’s hormonal pool (Cohen et al., 2009; Shaffique et al., 2024). In addition to direct hormone production, PGPR may stimulate endogenous ABA biosynthesis in plants by enhancing the expression of 9−cis−epoxycarotenoid dioxygenase (NCED), a key rate-limiting enzyme in ABA production. Upregulation of *NCED* results in elevated ABA accumulation in plant tissues, further strengthening drought-responsive signaling networks (Estrada-Melo et al., 2015).

Auxins and cytokinins are fundamental regulators of plant growth and development, particularly in maintaining the balance between root and shoot architecture (Kurepa and Smalle, 2022). Certain beneficial microorganisms synthesize and secrete these phytohormones, thereby reshaping RSA and promoting plant growth under stress conditions. Genomic analysis of *Bacillus velezensis* S141, a strain evaluated in cannabis, revealed the presence of *yhcx*, *IPyAD*, and *dhaS*, key genes in the tryptophan−dependent indole−3−pyruvic acid (IPyA) pathway responsible for auxin biosynthesis (Aunkam et al., 2024). This strain also contains isopentenyl transferase-related genes involved in cytokinin biosynthesis or precursor supply, indicating its capacity to directly modulate hormonal signaling in stressed cannabis plants (Aunkam et al., 2024). Under drought stress, plants typically accumulate ABA, which promotes stomatal closure to reduce transpirational water loss. However, microbially derived cytokinins can partially antagonize ABA signaling, preventing excessive stomatal closure that would otherwise suppress photosynthesis, while also retarding chlorophyll degradation. By moderating this hormonal balance, microbial cytokinins help sustain basal photosynthetic activity during drought, and facilitate more rapid physiological recovery when favorable conditions are restored (Aunkam et al., 2024).

Jasmonic acid (JA) and salicylic acid (SA) are traditionally recognized for their roles in plant defense against pathogens. However, accumulating evidence indicates that both hormones also contribute substantially to drought tolerance (Ku et al., 2018). Beneficial microorganisms, including *Pseudomonas* spp., can enhance the expression of *MYC2*, a central transcription factor in the JA signaling pathway. Activation of *MYC2* promotes downstream expression of genes such as *LOX2* and *VSP2*, which contribute to the regulation of stomatal conductance and the reduction of transpirational water loss (Pozo et al., 2008). In parallel, the SA signaling pathway modulates antioxidant defense systems and the fine control of stomatal aperture under drought conditions. Microbial inoculation has been shown to induce expression of the SA biosynthetic gene *ICS1* and the signaling regulator NPR1, collectively enhancing the activity of antioxidant enzymes that mitigate the accumulation of ROS generated during water deficit (Elsisi et al., 2024). Moreover, NPR1 functions as a key regulatory node in SA and JA crosstalk. Through interaction with TFs such as WRKY70, NPR1 facilitates dynamic coordination between these pathways, enabling plants to mount a balanced and finely regulated response to drought stress (Li et al., 2004; Rivas-San Vicente and Plasencia, 2011).

### 6.3 Aquaporins and microbially mediated transcriptional regulation under drought

Beyond inducing physiological and metabolic adjustments, beneficial microorganisms enhance drought resilience through coordinated transcriptional regulation of stress−responsive genes. In drought−stressed cannabis inoculated with *T. hamatum*, transcript levels of key drought-associated markers, including genes involved in proline and branched−chain amino acid (BCAA) biosynthesis, were restored to levels statistically indistinguishable from those observed in unstressed controls (Ryu et al., 2025). This pattern suggests that *T. hamatum* alleviates drought stress at a systemic level rather than relying solely on sustained accumulation of osmolytes such as proline or BCAA.

Aquaporins, membrane channel proteins that facilitate transcellular water transport across cell membranes, represent critical regulatory nodes in plant drought responses (J. Li et al., 2015). In maize, inoculation with *B. velezensis* upregulated aquaporin genes such as *ZmPIP2−6* and *ZmTIP1−1*, enhancing root hydraulic conductivity and mitigating drought effects (Zhang et al., 2026). Similarly, in trifoliate orange (*Poncirus trifoliata* L.), colonization by the AMF *F. mosseae* increased expression of *PtTIP1−2*, *PtTIP1−3*, and *PtTIP4−1*, correlating with improved drought tolerance (Jia-Dong et al., 2019). In cannabis, *T. hamatum* treatment resulted in upregulation of more than 90% of significantly responsive aquaporin genes, consistent with previous findings that enhanced aquaporin activity underlies microbially mediated drought adaptation (Ryu et al., 2025) (**Figure 7B**).

Beyond water transport, beneficial fungi influence structural resilience through transcriptional control. In cannabis, PGPF markedly induced expression of an L10−interacting MYB domain−containing protein gene, with an approximately 208−fold increase (log_2_ fold−change 7.70) (Ryu et al., 2025). MYB transcription factors constitute one of the largest regulatory families in plants and, in coordination with enzymes such as HCT, promote lignin biosynthesis, thereby reinforcing cell walls and reducing the risk of tissue collapse under water deficit (Li et al., 2020). MYB−mediated regulation also enhances cuticular wax deposition, which limits non−stomatal water loss (S. B. Lee et al., 2016). Collectively, these findings indicate that beneficial soil microorganisms enhance drought tolerance not only through biochemical modulation but also by strengthening the structural integrity of plant tissues.

A growing body of work has identified multiple microbial taxa that promote growth and alleviate drought stress in cannabis, including PGPR such as *Bacillus* (Aunkam et al., 2024); PGPF such as *Trichoderma* (Ryu et al., 2025); and AMF including *Rhizophagus* (Sun et al., 2022), *Glomus* and *Funneliformis* (Msairi et al., 2023). Nevertheless, the exogenous application of a single microbial strain or consortium does not guarantee consistent outcomes across diverse cultivation systems and cannabis genotypes. Plants actively shape and select their rhizosphere microbiome through dynamic modulation of root exudate composition, reflecting their physiological status and environmental context (Barnes et al., 2025).

## 7 Future perspectives and research directions

Although cannabis exhibits relatively high WUE and considerable physiological adaptability, the interplay among stomatal plasticity, ABA signaling, ROS homeostasis, and hydraulic regulation remains incompletely understood (Cutler et al., 2010; Kim et al., 2010; Haworth et al., 2024). This knowledge gap limits mechanistic understanding of how cannabis balances water conservation with carbon assimilation and sustains physiological recovery following drought exposure. In addition, the genetic basis underlying resilience to repeated drought cycles and recovery after rewatering has received limited attention. Integrating physiological observations with molecular and genetic analyses may help explain how coordinated stress responses influence drought adaptation and performance in cannabis.

The contribution of root systems to drought adaptation in cannabis is likewise insufficiently characterized, largely due to the technical challenges associated with root phenotyping under soil-based conditions. As a consequence, understanding of how RSA contributes to water acquisition efficiency, hydraulic stability, and drought tolerance in cannabis is still limited (Siddiqui et al., 2021; Schneider et al., 2022). Although homologs of major RSA-associated genes, including *DRO1*, *Rt1*, and *NRT* family members, have recently been identified through in silico analyses, their functional roles and regulatory interactions under water-deficit conditions have yet to be experimentally validated (Morales et al., 2026). This gap constrains the identification of drought-adaptive root traits and limits the translation of RSA-associated variation into breeding strategies. Likewise, the coordinated roles of auxin signaling, transcription factor networks, root anatomical plasticity, and root-to-shoot communication in drought adaptation are only beginning to be explored in cannabis. Integrating high-throughput root phenotyping with genomic and field-based validation approaches will therefore facilitate the identification of drought-adaptive root ideotypes that can be incorporated into breeding programs to improve water acquisition efficiency, drought resilience, and yield stability in cannabis cultivation systems.

Understanding how water-deficit stress influences cannabinoid production and overall productivity remains another important challenge in cannabis research. A major limitation across existing studies is the inconsistent or absent quantification of key physiological parameters, particularly stomatal conductance and leaf water potential, which are essential for accurately defining drought severity. As a result, reported increases in THC or CBD concentrations under different levels of water-deficit stress are frequently misinterpreted as improvements in overall productivity, even though such increases often coincide with reductions in floral biomass and total cannabinoid yield per plant (Allred et al., 2025). Furthermore, responses may vary across developmental stages, yet stage-specific assessments during flowering remain limited regarding the optimal timing of stress application.

Mechanistic understanding is further hindered by the scarcity of studies examining trichome developmental dynamics, cannabigerolic acid (CBGA) flux, and the regulation of THCA and CBDA synthase gene expression under well-controlled drought conditions. To improve consistency and reproducibility, future studies should adopt standardized and clearly defined water-deficit protocols supported by routine measurements of leaf water potential, stomatal conductance, and soil moisture, and canopy temperature, to improve cross-study comparability and establish reproducible benchmarks for drought phenotyping in cannabis. Investigating water limitation across multiple flowering stages would also help identify periods of heightened sensitivity and clarify developmental plasticity in cannabinoid metabolism. Distinguishing between changes in cannabinoid concentration and total yield will be essential to avoid misleading interpretations of drought effects.

Additionally, mechanistic insight would benefit from targeted evaluations of trichome morphogenesis and the regulatory genetics of cannabinoid biosynthesis under controlled stress gradients. Integrating metabolomic profiling with transcriptomics and gene-expression analyses would substantially improve understanding of how water-deficit stress reshapes primary and secondary metabolic networks. Furthermore, a more holistic framework considering interactions among water availability, nutrient supply, light intensity, and other environmental variables will be crucial for understanding how multiple factors collectively influence cannabis growth, physiology, and phytochemical output.

Understanding how drought-responsive regulatory networks are coordinated at the post-transcriptional and epigenetic levels remains an important challenge in cannabis research. Although drought-responsive miRNAs, MYB transcription factors, and APX gene family have recently been identified in cannabis, relatively little is known about their temporal regulation, tissue specificity, and downstream interactions under prolonged drought conditions (Liang et al., 2024; Wang et al., 2026). This knowledge gap complicates interpretation of how ncRNAs, ABA signaling, transcription factor activity, and oxidative stress responses collectively shape drought adaptation and stress memory in cannabis. In addition, much of the current framework is still inferred from model and crop species, while cannabis-specific studies remain limited (Bolc et al., 2025). The potential contributions of lncRNAs and circRNAs are particularly poorly understood despite growing evidence supporting their involvement in abiotic stress adaptation across plants (Bao et al., 2025; Kumar Saroha et al., 2026). Integrating transcriptomics, epigenomics, and small RNA profiling should be prioritized to identify regulatory biomarkers that can accelerate molecular breeding and improve drought resilience in future cannabis cultivars.

Beyond molecular regulation, drought adaptation in cannabis is also shaped by biotic interactions within the rhizosphere, particularly plant-microbe symbioses that influence water acquisition, nutrient availability, and stress signaling (Dong et al., 2019). Despite growing interest in the role of beneficial microorganisms in improving stress resilience, microbe-mediated drought adaptation in cannabis remains insufficiently characterized. Current evidence suggests that the effectiveness of microbial inoculants is highly dependent on cultivar specificity, soil properties, and environmental conditions, indicating that generalized inoculation strategies are unlikely to provide consistent outcomes across cultivation systems. Developing reliable microbial applications will require cultivar-specific validation and multi-environment testing to enable their practical adoption in commercial cannabis production systems. Several important limitations currently restrict progress in this area. Most studies investigating PGPR, PGPF, and AMF-mediated drought tolerance in cannabis are limited to a small number of cultivars and frequently rely on simplified short-term greenhouse stress models (Pagnani et al., 2018; Yuan et al., 2024). In addition, the predominant focus on single-strain inoculations and endpoint physiological measurements provides only limited insight into microbial community interactions and the mechanistic basis of stress alleviation. Future research should therefore integrate multi-omics, microbiome profiling, and functional physiological analyses to characterize the dynamic regulatory networks underlying microbe-mediated drought adaptation. Expanding field-based studies across diverse environmental conditions will also be essential for developing stable and scalable microbial strategies for sustainable cannabis cultivation.

Improving cannabis performance under diverse environmental conditions will require integrated advances in genetics, breeding, and precision cultivation strategies. The development of molecular markers could improve breeding efficiency by enabling early-stage screening and the selection of superior genotypes (Soler et al., 2017; Borin et al., 2021; Delpasand Khabbazi, 2025; Khabbazi, 2025, 2026). Genome editing and genetic engineering tools may also facilitate the development of new varieties with improved agronomic traits, stress tolerance, and secondary metabolite profiles (Bao et al., 2019; Xu et al., 2019; Khabbazi et al., 2024). However, the effective application of these approaches will depend on a clearer understanding of regulatory networks, secondary metabolism, and the physiological trade-offs influencing plant growth and productivity. Future breeding programs should integrate drought-resilient root system architecture, water-use efficiency, and stable cannabinoid yield as simultaneous selection targets rather than optimizing these traits independently. Likewise, standardized drought phenotyping platforms combined with physiological indicators such as leaf water potential, stomatal conductance, and canopy temperature should be incorporated into breeding pipelines to improve selection accuracy and reproducibility under water-limited environments. From a cultivation perspective, integrating precision irrigation technologies, real-time plant and soil sensors, and data-driven irrigation scheduling could enable dynamic water management strategies that maximize water-use efficiency while maintaining floral biomass and cannabinoid productivity. Furthermore, validating drought-adaptive genotypes and cultivation practices under diverse environmental conditions will be essential for translating experimental findings into commercially applicable production systems. Collectively, these advances will accelerate the development of climate-resilient cannabis cultivars and support more sustainable production systems capable of maintaining productivity and cannabinoid quality under increasing water scarcity.

## 8 Conclusions

Drought adaptation in cannabis involves coordinated changes in photosynthetic performance, photoprotective capacity, osmotic adjustment, and water-use behavior, with stomatal plasticity emerging as a key mechanism for balancing carbon assimilation and transpirational water loss under water-limited conditions. In addition, this adaptation is also influenced by belowground traits and phytochemical adjustments, although their contribution is still not fully understood. Drought has also been associated with changes in glandular trichome development and phytochemical accumulation.

At the molecular level, RNA-mediated and epigenetic regulation of drought responses have received limited attention in cannabis, leaving important questions regarding stress adaptation unresolved. In addition to these endogenous regulatory mechanisms, beneficial rhizosphere microorganisms may contribute to drought resilience through metabolic and physiological reprogramming. Overall, drought adaptation in cannabis should be considered a multi-layered and highly integrated process requiring coordinated investigation across physiological, molecular, and rhizosphere-associated dimensions. Advancing this understanding will be essential for improving cannabis performance and resilience under increasingly water-limited cultivation systems.
